# Visual Quantitation of Copper Ions Based on a Microfluidic Particle Dam Reflecting the Cu(II)-Catalyzed Oxidative Damage of DNA

**DOI:** 10.3390/bios11120487

**Published:** 2021-11-30

**Authors:** Chenyu Cui, Ting-Hsuan Chen

**Affiliations:** Department of Biomedical Engineering, City University of Hong Kong, 83 Tat Chee Avenue, Hong Kong, China; chenyucui2-c@my.cityu.edu.hk

**Keywords:** copper ion, Fenton reaction, microfluidics, visual detection

## Abstract

Due to the use of copper water pipes and the discharge of industrial wastewater, contamination of copper ions in drinking water has become a severe hazard globally. To routinely check water safety on a daily basis, easy-to-use platforms for quantitative analysis of trace amounts of copper ions (Cu^2+^) in drinking water is needed. Here, we report microfluidic particle accumulation integrated with a Cu(II)-catalyzed Fenton reaction for visual and quantitative copper ion detection. Microparticles (MMPs) and polystyrene microparticles (PMPs) are connected via a single strand DNA, MB155. However, when Cu^2+^ is present, MB155 is cleaved by hydroxyl free radicals (•OH) produced from Cu^2+^/hydrogen peroxide (H_2_O_2_) Fenton reactions, causing an increased amount of free PMPs. To visually count them, the particle solution is loaded onto a microfluidic chip where free MMPs and MMPs–MB155–PMPs can be collected by the magnetic separator, while the free PMPs continue flowing until being accumulated at the particle dam. The results showed a good linear relationship between the trapping length of PMP accumulation and the Cu^2+^ concentration from 0 to 300 nM. A limit of detection (LOD) of 70.1 nM was achieved, which is approximately 449 times lower than the 2 × 10^3^ μg·L^−1^ (~31.5 μM) required by the World Health Organization (WHO). Moreover, the results showed high selectivity and good tolerance to pH and hardness, indicating compatibility for detection in tap water, suggesting a potential platform for the routine monitoring of copper contamination in drinking water.

## 1. Introduction

Cu^2+^ is an essential element participating in many biological systems such as the central nervous system [1], the immune system [2], and the functioning of internal organs such as the heart [3]. However, an excess amount of Cu^2+^ may burden the organs and lead to illnesses such as liver and kidney dysfunction [4,5] and neurodegenerative diseases such as Alzheimer’s and Parkinson’s diseases [6,7]. Unfortunately, because of the increased industrialization and urbanization, the metal deposits from manufacturing byproducts accumulate in the soil and sediments of water bodies, which cannot be completely removed by the existing tap water treatment process and could be even worsened after pipeline transportation. Ultimately, excess ions enter the food chain and seriously affect human health. To avoid this, a maximum level of copper in drinking water was set to 2.00 × 10^3^ μg·L^−1^ (~31.5 μM) by the WHO [8], which is used in the routine analysis and monitoring of trace amounts of Cu^2+^ in drinking water to ensure water safety.

The traditional detection of Cu^2+^ relies on atomic absorption spectroscopy (AAS) [9,10,11], inductively coupled plasma mass spectroscopy (ICP-MS) [12], and high-performance liquid chromatography (HPLC) [13], where bulky equipment, complicated sample preparation, and long testing times are inevitable. To simplify the process, many analytical methods have been developed based on colorimetry [14,15,16], electrochemistry [17,18], and fluorescence [19,20]. Among them, some researchers have achieved Cu^2+^ detection based on the oxidation-reduction property of Cu^2+^, which can produce hydroxyl radicals [21,22]. Zhao et al. [23] used silicon quantum dots (SiQDs) with fluorescence for the detection of Cu^2+^. When Cu^2+^ is present, the fluorescence is quenched by hydroxyl radicals generating from the Fenton reaction between H_2_O_2_ and Cu^+^, resulting in a decrease in the fluorescence intensity. However, such a method relies on the fluorescence spectrophotometer, which is still laboratory-based and not user-friendly. In contrast, Wang et al. [24] developed a lateral flow test strip for Cu^2+^ detection based on Cu^+^-catalyzed click chemistry and hybridization of single-stranded DNA, where the presence of Cu^2+^ can be visualized by hybridization of ssDNA-labeled gold nanoparticles. However, lateral flow strips can hardly achieve quantitative measurement and mostly yield only yes/no results.

**Figure 1:**
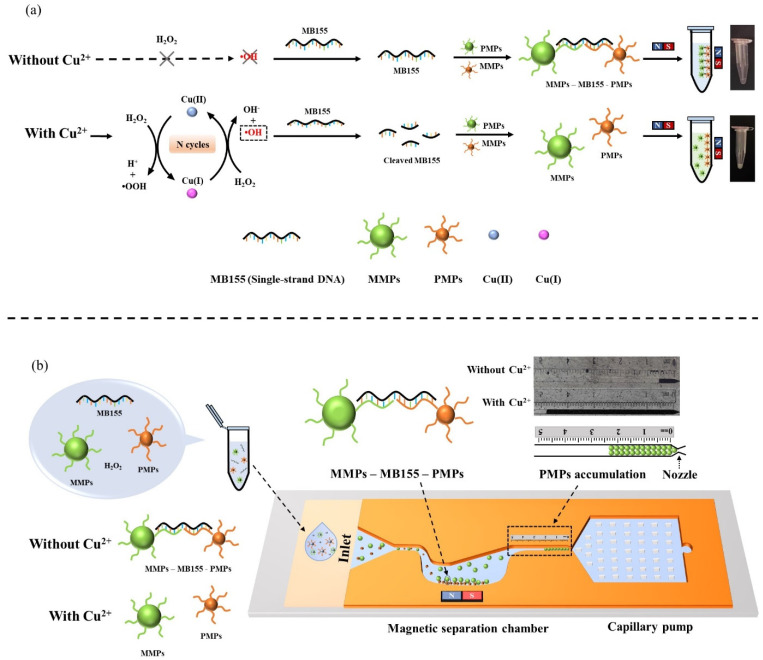
Working principle of the visual quantitative detection of Cu^2+^. (**a**) MB155 connects MMPs and PMPs through DNA hybridization, resulting in a clear solution after magnetic separation. In contrast, the presence of Cu^2+^ in H_2_O_2_ generates •OH, which cleaves MB155. As such, microparticles are disconnected, making the solution turbid. (**b**) After reaction, the particle solution is loaded onto a microfluidic chip, where free MMPs and MMPs–MB155–PMPs are separated by a magnet, and the free PMPs continue flowing until being trapped and accumulated at the particle dam, which is narrow in width, enabling the quantitative determination of Cu^2+^ by visual inspection.

In this work, we report a visual and quantitative method for detecting Cu^2+^ on a microfluidic platform (Figure 1). Cu^2+^, the analyte, is first reduced to Cu^+^ by H_2_O_2_, and the produced Cu^+^ subsequently reacts with H_2_O_2_ using the Fenton reaction, generating •OH that randomly fragmentizes single-strand DNA oligonucleotides through oxidative damage. Next, Cu^+^ is oxidized to Cu^2+^, forming a reaction cycle that allows signal amplification. To visualize the DNA damage, MB155, a single-strand DNA, is used to connect MMPs and PMPs through DNA hybridization, forming a sandwich structure, MMPs–MB155–PMPs. In contrast, with the presence of Cu^2+^, the cleavage of MB155 induced by •OH leads to separation of MMPs and PMPs. To visualize the altered particle connection, the particle solution is loaded onto a microfluidic chip containing a magnetic separator to remove the free MMPs and MMPs–MB155–PMPs, leaving the PMPs to continue flowing until being trapped and stacked at the trapping channel. As a result, the trapping length of PMP accumulation, which is similar to a thermometer-like display and proportional to the concentration of Cu^2+^, can be easily read and interpreted by the naked eye. After obtaining the LOD and exploring the selectivity and tolerance to environmental interferences, it was applied to detect Cu^2+^ in tap water, demonstrating the potential for monitoring Cu^2+^ contamination in daily drinking water.

## 2. Materials and Methods

### 2.1. Preparation of Oligonucleotides

The sequences of P1, P2, and MB155 (BGI BIO-Solutions HONGKONG Co., Ltd., Hong Kong, China) used in this work are shown in Table S1. P1 and P2 were designed with sequences complementary to that of MB155 in juxtaposition. Both P1 and P2 with biotin modification for binding to streptavidin-modified microparticles were purified using high-performance liquid chromatography, while MB155 was purified by polyacrylamide gel electrophoresis. The powdered oligonucleotides received from the suppliers were dissolved in nuclease-free water (Thermo Fisher Scientific, Waltham, MA, USA) to obtain a stock solution of 100 μM and stored at 4 °C for further use.

### 2.2. Cu(II)-Catalyzed Fenton Reaction

For Cu^2+^ detection, 2 μL of MB155 (5 μM for magnetophoresis assay or 3.75 μM for microfluidic chip) and 2 μL of 5 M of H_2_O_2_ (Anaqua) were mixed with 20 μL of 50 mM Tris-HCl buffer (pH = 7.4). Next, 76 μL of different concentrations of Cu^2+^ diluted in DI water was added and reacted for 30 min at room temperature. For the selectivity experiment, the 76 μL Cu^2+^ solution was replaced by different metal ions—cadmium (Cd^2+^), Barium (Ba^2+^), mercury (Hg^2+^), zinc (Zn^2+^), manganese (Mn^2+^), strontium (Sr^2+^), lead (Pb^2+^), and iron (Fe^2+^) (J&K Scientific, Hong Kong). For testing the pH, Cu^2+^ of different pH values (6.0, 6.5, 7.0, 7.5, and 8) were prepared by adding HCl or NaOH. For testing the water hardness, various brands of bottled water with different water hardnesses were used for diluting Cu^2+^. Here, soft water (Bourbon^TM^) with a hardness of 55.0 mg/L, moderately hard water (FIJI^TM^) with a hardness of 108 mg/L, hard water (AQUA^TM^) with a hardness of 158 mg/L, and very hard water (Vuttel^TM^) with a hardness of 318 mg/L were chosen. For detection in tap water, 76 μL of tap water was used.

### 2.3. Agarose Gel Electrophoresis

Five grams of agarose powder (Thermo Fisher Scientific, Waltham, MA, USA) was mixed with 10 mL of 10× Tris-Acetate-EDTA (TAE) buffer (Thermo Fisher Scientific, Waltham, MA, USA) and 90 mL of deionized (DI) water (Milli-Q Plus system, with a resistivity of 18.2 MΩ cm), followed by heating in a microwave for 2 min to fully dissolve the agarose. Next, 3 μL of 10,000 × GelRed^®^ Nucleic Acid Gel Stain (Biotium, Fremont, CA, USA) was added, followed by heating for another 1 min. Then, the dissolved agarose was poured into a tray with a well comb in place immediately. After cooling down for 30 min, the solidified gel was placed into the gel box and covered by 1× TAE buffer. An ultra-low range DNA ladder (SM1211, Thermo Fisher Scientific, Waltham, MA, USA) was 5 times diluted and then loaded into the first lane of the gel. Then, 10 μL of the reaction mixture containing 2 μL of 5 μM MB155, 2 μL of 5 mM H_2_O_2_, 2 μL of 5 mM Cu^2+^, 2 μL of 50 mM Tris-HCl buffer, and 2 μL of DI water was mixed with 2 μL of 6× DNA loading dye (Thermo Fisher Scientific, Waltham, MA, USA) and loaded into the additional wells of the gel. After running at 120 V for 60 min, a gel image was obtained using a BIO-RAD Gel Doc EZ Imager.

### 2.4. Magnetophoresis Assay

First, 3.5 μL of MMPs (CME0101, 0.86 μm in diameter, streptavidin-coated, 1.827 × 10^10^ microspheres/mL, Bangs Laboratories, Inc., Fishers, IN, USA) was mixed with 3.5 μL of biotin-modified P1, while 2.5 μL of PMPs (CFDG004, 0.955 μm in diameter, streptavidin-coated, 2.07 × 10^10^ microspheres/mL, Bangs Laboratories, Inc., Fishers, IN, USA) were mixed with 2.5 μL of biotin-modified P2. The mixture was shaken at room temperature for 30 min to obtain MMPs–P1 and PMPs–P2 complexes through streptavidin–biotin bonds. Then, the excess probe was removed by rinsing with washing buffer (50 mM Tris-HCl, 150mM NaCl, pH = 7.4, 0.2% Tween 20, pH 7.5) three times. During this step, a magnetic rack was applied for 1 min to collect MMPs, while for the separation of PMPs, a centrifuge at 13.3× *g* was conducted for 2 min. After washing, the particle solution was brought back to its original volume. Then, 10 μL of the resulting solution from the Cu^2+^-induced Fenton reaction was extracted and added to a mixture of 3.5 μL of MMPs–P1, 2.5 μL of PMPs–P2, and 4 μL of washing buffer, followed by 30 min of shaking for the formation of MMPs–MB155–PMPs. After placing the solution on a magnetic rack to collect the separated MMPs and the complex of MMPs–MB155–PMPs, the turbidity of supernatant was observed by the naked eye, or measured by the optical absorbance at 365 nm by UV-Vis spectrometer (BioDrop μLITE, Cambridge, UK).

### 2.5. Detection on the Microfluidic Chip

The fabrication process of the chip is shown in the Supplementary Materials. For detection on the chip, MMPs (0.32 µm diameter, SVM-025-5H, streptavidin-coated, 5 mg/mL, Spherotech. Inc., Lake Forest, IL, USA) were washed three times by MES buffer (100 mM MES, 0.1% BSA, 0.1% Tween 20, pH 4.5) and incubated in MES buffer for 1 h with the original volume. After that, 5 μL of MMPs was mixed with 5 μL of 100 μM P1, while 5 μL of PMPs (15.3 µm diameter, CP01008, streptavidin-coated, 5.033 × 10^6^ microspheres mL^−1^, Bangs Laboratories, Inc., Fishers, IN, USA) was mixed with 5 μL of 100 μM P2, followed by shaking for 30 min. The same washing steps as that for the magnetophoresis assay were used, as follows. Before loading to the chip, 5 μL of the resulting solution after the Cu^2+^-induced Fenton reaction, as mentioned, was extracted and mixed with 5 μL of MMPs–P1 and 5 μL of PMPs–P2 with gentle shaking for 30 min. Finally, 3 μL of the final solution was loaded at the microfluidic chip’s inlet and flowed into the channel based on capillary force. The free MMPs and MMPs–MB155–PMPs were attracted to the bottom of the magnetic separator of the chip, while the non-connected PMPs with a diameter of 15.3 µm were blocked at the trapping channel with an 8 µm wide particle dam. The trapping length after PMP accumulation was inspected by the naked eye and recorded by a microscope.

## 3. Results and Discussion

### 3.1. Agarose Gel Electrophoresis of the Cleaved ssDNA

**Figure 2:**
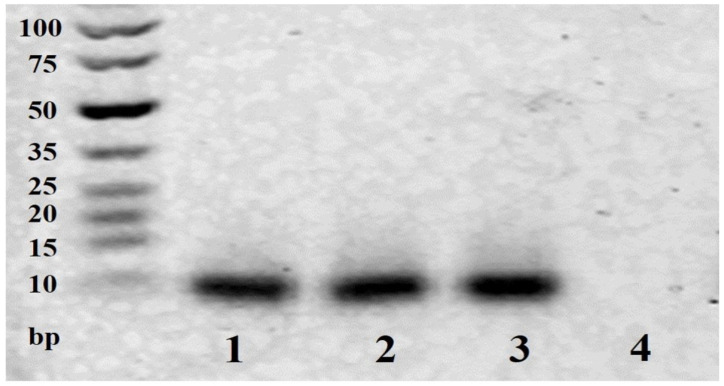
Agarose gel of MB155 cleavage. (Lane 1: MB155; Lane 2: MB155 + H_2_O_2_; Lane 3: MB155 + Cu^2+^; Lane 4: MB155 + H_2_O_2_ + Cu^2+^). Concentration (MB155) = 1 μM. Concentration (H_2_O_2_ and Cu^2+^) = 1 mM.

Agarose gel was used to identify the base damage of MB155 by H_2_O_2_ in the presence of Cu^2+^ (Figure 2). As shown in Lanes 2 and 3, H_2_O_2_ or Cu^2+^ alone did not cause noticeable cleavage of MB155, but the band of MB155 disappeared when Cu^2+^ and H_2_O_2_ coexisted (Lane 4), which demonstrates the efficient cleavage of MB155 induced by •OH generated from H_2_O_2_ and Cu^2+^.

### 3.2. Magnetophoresis Assay

The magnetophoresis assay was first used to assess the altered connection between MMPs and PMPs upon the cleavage of MB155 induced by the Cu^2+^/H_2_O_2_ system. The MMPs–MB155–PMPs complex was formed due to the hybridization of MB155 with P1 and P2 modified on microparticles. When applying a magnetic force, MMPs and MMPs–MB155–PMPs were pulled onto the tube wall, leaving non-connected PMPs suspending in the solution (Figure 1). Thus, when there was no Cu^2+^, almost all of the PMPs were in the form of MMPs–MB155–PMPs, which could be separated from the solution after magnetic attraction, resulting in a clear solution. On the contrary, adding Cu^2+^ would make the solution turbid due to the Mie scattering from increased free PMPs, which could be measured by the relative optical absorbance at 365 nm of the supernatant with PMPs.

With a magnetophoresis assay to quantify the particle connection, different concentrations of MB155 and H_2_O_2_ and reaction times were optimized (Figure S1). First, 50 nM of MB155 (in the final solution containing Cu^2+^, H_2_O_2_, MMPs, and PMPs) was chosen as the optimal concentration because of its highest signal-to-noise ratio (Figure S1a). For optimization of the H_2_O_2_ concentration, 10 mM of H_2_O_2_ was inadequate for reacting with trace amounts of Cu^2+^. However, 1000 mM of H_2_O_2_ may cause undesired damage to particle connections, as seen by the high absorbance value of the blank control. Therefore, 100 mM of H_2_O_2_ was selected (Figure S1b). The reaction time for Cu^2+^ and H_2_O_2_ was also optimized as 30 min, as it achieved the ideal low background and was still sufficient to generate •OH to cleave the MB155 (Figure S1c).

**Figure 3:**
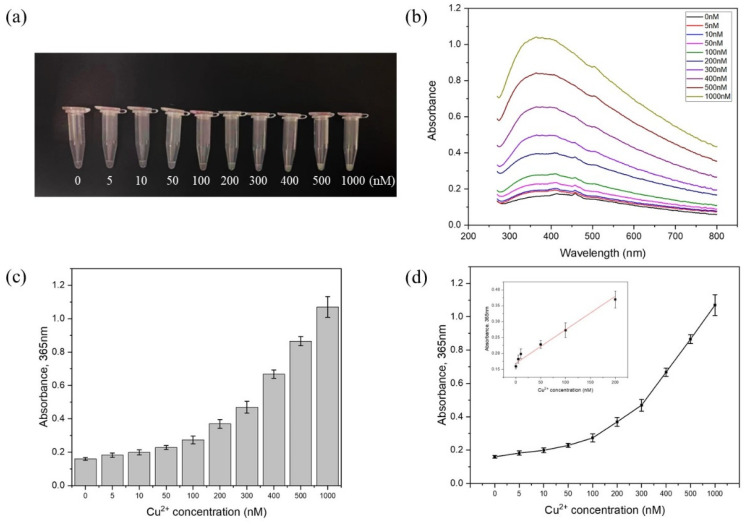
Magnetophoresis assay for Cu^2+^ detection. (**a**) Optical image showing the change in the turbidity of the solution from clear to turbid with the increasing concentration of Cu^2+^. (**b**) Absorbance spectrum of solutions under different concentrations of Cu^2+^. (**c**) Absorbance value (mean ± maximum deviation, *n* = 3) at 365 nm corresponding to (**a**). (**d**) Linear regression of the absorbance value versus Cu^2+^ concentration.

After optimizing the experimental conditions, we explored the limit of detection under a series of Cu^2+^ concentrations (0 nM, 5 nM, 10 nM, 50 nM, 100 nM, 200 nM, 300 nM, 400 nM, 500 nM, and 1000 nM). The turbidity of the extracted suspension after magnetic separation became opaquer with the increment in the Cu^2+^ concentration (Figure 3a). In addition, the difference could be visually distinguished when the Cu^2+^ concentration reached 50 nM. To obtain more accurate quantification, a UV-Vis spectrometer was applied to measure the optic absorbance spectrum corresponding to the Mie scattering effect caused by free PMPs (Figure 3b). The results showed an increment in the absorbance value accompanying the increase in the Cu^2+^ concentration. Moreover, there was a significant linear relationship between the Cu^2+^ concentration and absorbance within the range of 0–200 nM. The linear regression equation was $y=0.000975x+0.176\pm 0.0301{\left[\frac{7}{18}+\frac{{\left(x-60.8\right)}^{2}}{6.82\times 10^{5}}\right]}^{\frac{1}{2}}(R^{2}=0.985)$, where *x* represents the concentration of Cu^2+^ and *y* means the absorbance value. The value of *S*_b1_ (the uncertainty of the slope) and *S*_b0_ (the uncertainty of the intercept) were 6.07 × 10^−5^ and 0.00568, respectively (Figure 3d). Thus, the LOD was 67.8 nM (see detailed calculation equations in the Supplementary Materials) [25,26].

### 3.3. LOD on the Microfluidic Chip Test

Next, considering the application for routine checking on a daily basis, we applied the microfluidic chip for visual and quantitative detection of Cu^2+^, such that the use of a UV-Vis spectrometer could be avoided. The H_2_O_2_ concentration and reaction time were based on the optimized results of the magnetophoresis assay. Here, because magnetic separation was carried out during capillary-driven particle flowing, smaller MMPs (0.32 µm in diameter) were used to minimize the drag force during magnetic separation. Additionally, the diameter of the PMPs was also changed to 15.3 µm, such that the particle dam with an 8 µm width could block the PMPs. Thus, the particle ratio and the MB155 concentration for optimal particle connection were further explored. At first, we used a 1:1 volume ratio of MMPs and PMPs to optimize the MB155 concentration to be 125 nM based on its shortest trapping length of PMP accumulation (Figure S2a). Next, the amount of MMPs was diluted while maintaining the same ratio of MMPs to MB155. The results showed that a 5× dilution of MMPs with 25 nM of MB155 was sufficient to connect the PMPs (Figure S2b).

**Figure 4:**
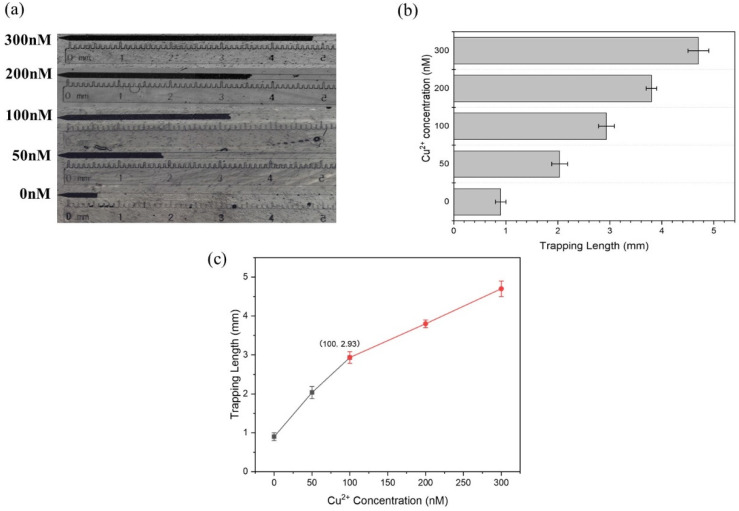
Cu^2+^ detection on the microfluidic chip. (**a**) Optical image showing the accumulating PMPs under different Cu^2+^ concentrations. (**b**) The trapping length (mean ± maximum deviation, *n* = 3) corresponding to (**a**). (**c**) The linear relationship between the trapping length and the Cu^2+^ concentration.

Next, the LOD was obtained by recording the trapping length with a varied concentration of Cu^2+^. The results showed an increasing trapping length when the Cu^2+^ concentration increased, which achieved visual quantitation of Cu^2+^ (Figure 4). Notably, the linear range was from 0 to 300 nM Cu^2+^ (Figure 3c), which could be fitted with linear regression Equation (1),

(1) $$y=0.020x+0.938\pm 0.597{\left[\frac{4}{9}+\frac{{\left(x-50.0\right)}^{2}}{7.26\times 10^{4}}\right]}^{\frac{1}{2}},(R^{2}=0.996,\;S_{b1}=0.00135,\;\mathrm{and}\;S_{b0}=0.0870)$$

for 0–100 nM of Cu^2+^ and Equation (2),

(2) $$y=0.00883x+2.04\pm 0.0236{\left[\frac{4}{9}+\frac{{\left(x-200\right)}^{2}}{2.18\times 10^{5}}\right]}^{\frac{1}{2}},\;(R^{2}=0.999,\;S_{b1}=9.62\times 10^{-5},\;\mathrm{and}\;S_{b0}=0.0208)$$

for 100–300 nM of Cu^2+^, where *y* represents the trapping length and *x* represents the concentration of Cu^2+^. Thus, the LOD could be determined as 70.1 nM according to Equation (1) (see detailed calculation equations from the Supplementary Materials) [25,26]. Considering the prescribed toxicity standard, i.e., ~30 µM in Hong Kong, ~20 µM from the US EPA (United States Environmental Protection Agency), and ~15 µM in mainland China, such an LOD was sufficiently low to monitor the toxicity level of the copper contamination in drinking water and comparable to other reported methods while achieving visual quantification (Table 1).

### 3.4. Tolerance to Environmental Interference

**Figure 5:**
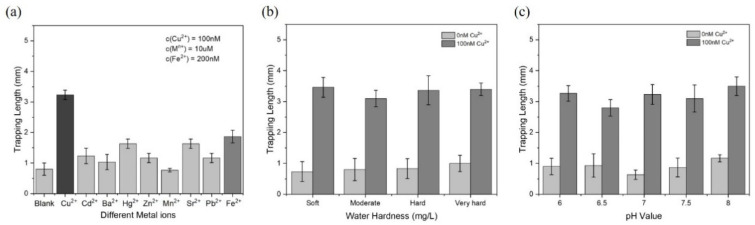
Tolerance of the proposed detection method to environmental interferences. (**a**) Selectivity in the presence of other metal ions (Cd^2+^, Ba^2+^, Hg^2+^, Zn^2+^ Mn^2+^, Sr^2+^, Pb^2+^, and Fe^2+^) with higher concentrations. (**b**). Detection of Cu^2+^ in environments with different water hardnesses (soft: 55.0 mg/L; moderately hard: 108 mg/L; hard: 158 mg/L; very hard: 318 mg/L). (**c**) Detection of Cu^2+^ in a pH range from 6.0 to 8.0 (mean ± maximumf deviation, *n* = 3).

The tolerance to other interferences was investigated to verify its compatibility with drinking water. We conducted the test under different common metal ions such as Cd^2+^, Ba^2+^, Hg^2+^, Zn^2+^ Mn^2+^, Sr^2+^, Pb^2+^, and Fe^2+^. As shown in Figure 5a, all of the metal ions with high concentrations (10 µM), except for Fe^2+^, hardly caused a significant increase in the trapping length. In contrast, Fe^2+^ was able to react with H_2_O_2_ and generate •OH based on the Fenton reaction. However, 200 nM of Fe^2+^ caused a slight increase in the trapping length, i.e., 1.7 mm, but the presence of 100 nM of Cu^2+^ led to a much longer accumulation of PMPs, i.e., 3.2 mm, suggesting a more active and stable generation of •OH by the Cu^2+^/H_2_O_2_ system [28]. Next, we tested the tolerance to water hardness by collecting water samples with different hardnesses. For detection of 100 nM copper ions, the trapping lengths were significantly longer than that of the blank control, which demonstrates a high stability of Cu^2+^ sensing in various water hardnesses (Figure 5b). For detecting Cu^2+^ in an acidic/basic medium, Cu^2+^ was added to water with a pH value from 6.0 to 8.0. The results showed that there was an obvious contrast in the trapping length between the sample with and without Cu^2+^, demonstrating a high tolerance to acidic or basic water environments (Figure 5c).

### 3.5. Detection of Cu^2+^ in Tap Water

Based on the high sensitivity and good environmental tolerance, we next applied the detection to a tap water sample as a practical application. Tap water is a common source of Cu^2+^ contamination in daily life because of the use of copper in water pipes, which may cause damage to health. We applied our detection method to measure the Cu^2+^ concentration in tap water. The tap water sample contained 393 nM of Cu^2+^, determined by ICP-MS. Remarkably, the trapping length was 3.5 ± 0.15 mm for detecting copper ions in a 2 × diluted tap water sample with a total 65 min assay response time, which was calculated as 165 ± 5.46 nM using the inverse regression of Equation (2). After multiplying the dilution factor, the concentration of Cu^2+^ in the tap water was determined as 330 ± 10.9 nM, suggesting an 84.0% accuracy rate for application to tap water.

## 4. Conclusions

This work presented an enzyme-free, convenient, and effective strategy for visualizing and quantifying Cu^2+^ based on single-strand DNA cleavage in the Cu^2+^/H_2_O_2_ system and the accumulation of PMPs as a thermometer-like display readable by the naked eye. The design showed excellent sensitivity with an LOD of 70.1 nM, which is much lower than the maximum contaminant level, i.e., 31.5 μM, in drinking water according to the WHO. In addition, the method demonstrated a high selectivity and good tolerance to water hardness and pH. More importantly, it can be applied to detect Cu^2+^ in actual tap water samples with high accuracy (84.0%), demonstrating its potential for controlling copper contamination in drinking water.

## Figures and Tables

**Table 1:** The comparison between this work and recently reported Cu^2+^ detection works.

Method	LOD	Linear Range	Quantification	Ref.
Fluorescence	0.15 µM	0.1–0.6 µM	Fluorescence spectra	[19]
Colorimetric	23 nM	0.1–10 μM	Absorption spectra	[21]
Fluorescence	115 nM	0.333–66.6 μM	Fluorescence spectra	[27]
Visualization	70.1 nM	0–300 nM	The naked eye	This work

## Supplementary Materials

The following are available online at https://www.mdpi.com/article/10.3390/bios11120487/s1, Table S1: The sequence of oligonucleotides used in this work; Figure S1: Optimization of (a) the MB155 concentration using 100 mM of H_2_O_2_, (b) the H_2_O_2_ concentration using 50 nM of MB155, and (c) the reaction time for the magnetophoresis assay based on relative absorbance at 365 nm; Figure S2: Optimization of the particle ratio for Cu^2+^ on-chip detection. (a) Optimization of the MB155 concentration based on a 1:1 ratio (v:v) of MMPs and PMPs. (b) Optimization of the MMP concentration by diluting the MMPs while maintaining the same ratio of MMPs to MB155, microfluidic chip fabrication, linear regression equation, limit of detection, and inverse regression.
