# Supplementary material for: Visual Quantitation of Copper Ions Based on a Microfluidic Particle Dam Reflecting the Cu(II)-Catalyzed Oxidative Damage of DNA
Source: Biosensors (Basel). 2021 Nov 30;11(12):487. doi: 10.3390/bios11120487 (PMC8699697; doi:10.3390/bios11120487)
Supplement: Supplementary file 1 [file biosensors-11-00487-s001.zip › biosensors-1453790-supplementary.pdf]

biosensors

MDPI

Supplementary Information

Visual Quantitation of Copper Ions Based on a Microfluidic Particle Dam Reflecting the Cu(II)-catalyzed Oxidative Damage of DNA

Chenyu Cui and Ting-Hsuan Chen *

Department of Biomedical Engineering, City University of Hong Kong, 83 Tat Chee Avenue, Hong Kong Special Administrative Region, China
* Correspondence: thchen@cityu.edu.hk

Table S1. The sequence of oligonucleotides used in this work.

Strand Name	Sequence
P1	5’-/biotin/ CCCCTATCACG-3’
P2	5’-ATTAGCATTAA-/biotin/-3’
MB155	5’-TTAATGCTAATCGTGATAGGGG-3’

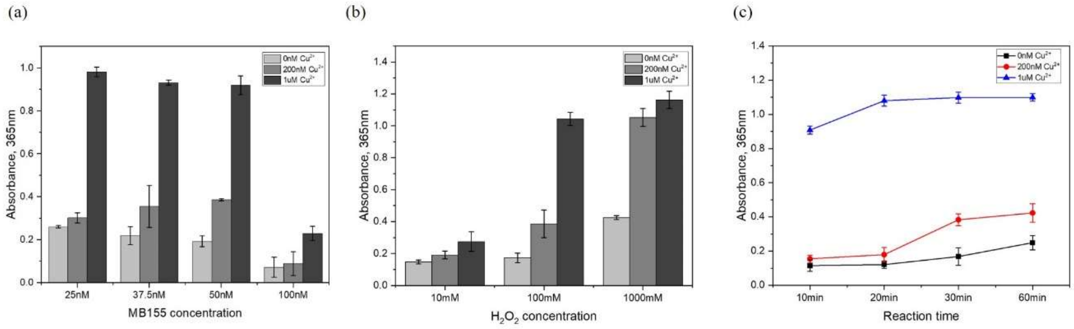

Figure S1. Optimization of (a) the MB155 concentration using 100 mM of H_2O_2, (b) the H_2O_2 concentration using 50 nM of MB155, and (c) the reaction time for the magnetophoresis assay based on relative absorbance at 365 nm.

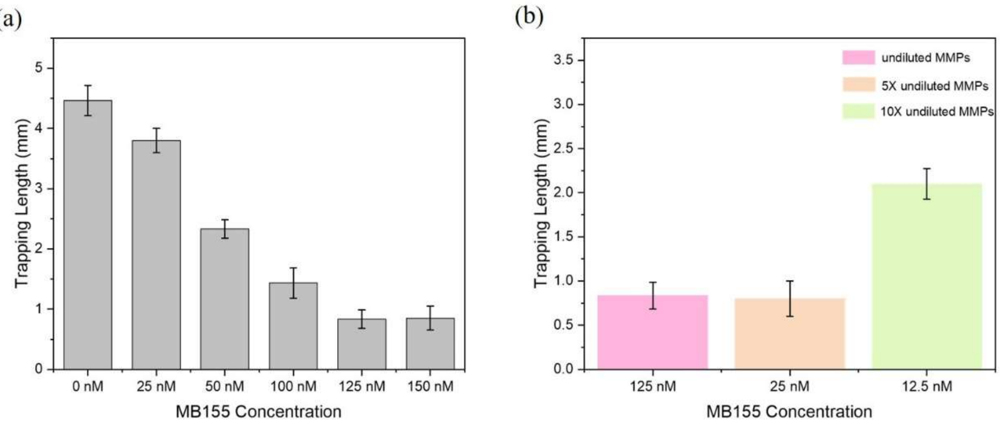

Figure S2. Optimization of the particle ratio for Cu^{2+} on-chip detection. (a) Optimization of the MB155 concentration based on a 1:1 ratio (v:v) of MMPs and PMPs. (b) Optimization of the MMP concentration by diluting the MMPs while maintaining the same ratio of MMPs to MB155.

Microfluidic Chip Fabrication

An SU-8 master (Gersteltec Sarl, Switzerland) with a 25 ± 0.3 μm thickness was fabricated by the photolithography process. Next, polydimethylsiloxane (PDMS; elastomer base/curing agent = 10:1, SylgardTM 184, Dow Corning, USA) was poured onto the SU-8 master, followed by oven curing at 70 °C for 2 h. Next, the PDMS was demolded and trimmed before 2 min of plasma treatment (Harrick Plasma, 400 mTorr) and coated with 3-Aminopropyl trimethoxysilane (Sigma Aldrich, USA) overnight in a vacuum chamber based on gas-phase deposition. A secondary PDMS casting was obtained using the first PDMS with a saline coating. Afterward, the secondary PDMS was peeled off and covered by NOA63, followed by a polypropylene film with a 100 μm thickness (PP, KOKUYO, Japan). Subsequently, the NOA63 with a pattern was solidified after 50 s of UV exposure and peeling off of the secondary PDMS. Next, a glass slide was treated with a 10 wt% trichloro (1H, 1H, 2H, and 2H-perfluorooctyl) silane (97%) (J&K Scientific Ltd.) coating for 40 min in the vacuum chamber. The patterned NOA63 sheet and a glass slide were then treated with 2 min of plasma treatment (Harrick Plasma, 800 mTorr) for bonding. During the plasma treatment, the sample loading area of the glass slide was covered by a tape to avoid plasma treatment so that the sample loading area could remain hydrophobic in contrast to the hydrophilic microchannels. At last, a magnet (size: 2.6 × 1.8 × 1.5 mm) was glued below the stomach-like structure of the microfluidic chip for magnetic separation.

Linear Regression Equation

Calculation of the calibration curve was carried out using the linear regression model of $y = b_0 + b_1 x$. The variables to achieve this calculation are outlined in the equations below.

$$\bar{x} = \sum_{i=1}^{n} \frac{x_i}{n} \qquad \text{Equation (1)}$$

$$\bar{y} = \sum_{i=1}^{n} \frac{y_i}{n} \qquad \text{Equation (2)}$$

$$b_0 = \bar{y} - b_1 \bar{x} \qquad \text{Equation (3)}$$

$$b_1 = \frac{\sum_{i=1}^{n} (x_i - \bar{x}) y_i}{\sum_{i=1}^{n} (x_i - \bar{x})^2} \qquad \text{Equation (4)}$$

$$s_{y/x}^2 = \frac{\sum_{i=1}^{n} (y_i - \hat{y}_i)^2}{n-2} \qquad \text{Equation (5)}$$

$$s_{b_0}^2 = s_{y/x}^2 \left(\frac{1}{n} + \frac{\bar{x}^2}{\sum_{i=1}^{n} (x_i - \bar{x})^2} \right) \qquad \text{Equation (6)}$$

$$s_{b_1}^2 = \frac{s_{y/x}^2}{\sum_{i=1}^{n} (x_i - \bar{x})^2} \qquad \text{Equation (7)}$$

where $\bar{x}$ and $\bar{y}$ are the average values of x and y, respectively; b_0 is the intercept; b_1 is the slope; and $s^2_{y/x}$, $s^2_{b_0}$, and $s^2_{b_1}$ are the variances of the y values, intercept b_0, and slope b_1. Meanwhile, n is the total measurement number, derived from $n = \sum_{j=1}^{k} m_j$, where j is the concentration level, k is the number of j, and m_j is the repeated testing times for every concentration level; $\hat{y}_i$ is the estimated y value for a specific concentration x_i obtained from $\hat{y}_i = b_0 + b_1 x_i$. Hence, we can achieve the final equation which describes the calibration curve as shown below.

$$\bar{y}_m^{\pm} = b_0 + b_1 x \pm t_{(1-\alpha/2, n-2)} s_{y/x} \left(\frac{1}{m} + \frac{1}{n} + \frac{(x-\bar{x})^2}{\sum_{i=1}^{n} (x_i - \bar{x})^2} \right)^{1/2} \quad \text{Equation (8)}$$

where $1-\alpha/2$ is the confidence interval of two-tailed hypothesis tests, $t_{(1-\alpha/2,\ n-2)}$ is the critical value of the Student's t distribution, and $1/m$ is the contribution of the uncertainty from the average of m replicates in future observations [1,2].

Limit of Detection

For the limit of detection calculation, it was obtained by the above calibration function and expressed as:

$$x_D = \delta_{(\alpha,\beta,n-2)} \frac{s_{y/x}}{b_1} \left(1 + \frac{1}{n} + \frac{\bar{x}^2}{\sum_{i=1}^{n} (x_i - \bar{x})^2} \right)^{1/2} \quad \text{Equation (9)}$$

where $\delta_{(\alpha,\ \beta,\ n-2)}$ is the non-centrality parameter protecting against type I and II errors (less than 5% of the positive or negative false rate) [2].

Inverse Regression

After observing the average trapping length on the chip, the estimated concentration of Cu^{2+} in tap water can be calculated by $\hat{x}_0 = (\bar{y}_{0m} - b_0)/b_1$, where $\bar{y}_{0m}$ is the average measurement value after repeated experiments. Thus, the variance of $\hat{x}_0$ is expressed as $s^2_{\hat{x}_0} = \frac{s^2_{y/x}}{b_1^2} \left(\frac{1}{m} + \frac{1}{n} + \frac{(\hat{x}_0 - \bar{x})^2}{\sum_{i=1}^{n} (x_i - \bar{x})^2} \right)$, where m represents the times of the repeated measurements.

Next, the inverse regression was obtained based on above calculation equation, which is shown as:

$$\hat{x}_0^{\pm} = \hat{x}_0 \pm t_{(1-\alpha/2, n-2)} s_{\hat{x}_0} \quad \text{Equation (10)}$$

where $t_{(1-\alpha/2, n-2)} s_{\hat{x}_0}$ is the estimated error and $t = 1.645$ for 90% of the confidence interval of two-tailed hypotheses [2].

References

1. Clayton, C.A.; Hines, J.W.; Elkins, P.D. Detection limits with specified assurance probabilities. *Anal. Chem.* **1987**, *59*, 2506–2514, doi:10.1021/ac00147a014.
2. Lavagnini, I.; Magno, F. A statistical overview on univariate calibration, inverse regression, and detection limits: Application to gas chromatography/mass spectrometry technique. *Mass Spectrom. Rev.* **2007**, *26*, 1–18, doi:10.1002/mas.20100.
